# Collaborative Supply Mechanism of Government-Subsidized Rental Housing from the Perspective of Tripartite Evolutionary Game in Metropolitan Cities of China

**DOI:** 10.1155/2022/4895099

**Published:** 2022-03-28

**Authors:** Xiaojun Liu, Jie Dong, Peng Cui, Mengmeng Wang, Xiaotong Guo

**Affiliations:** ^1^School of Management, Xi'an University of Architecture and Technology, Xi'an 710055, China; ^2^College of Civil Engineering, Nanjing Forestry University, Nanjing 210037, China; ^3^Laboratory of Neuromanagement in Engineering, Xi'an University of Architecture and Technology, Xi'an 710055, China

## Abstract

With the advancement of urbanisation, the inflow of population in China's large cities has been increasing and the demand for rental housing of “new citizens” with insufficient housing affordability has become increasingly strong. Therefore, the Chinese government proposes to provide government-subsidized rental housing (GSRH) different from public rental housing. At present, the supply mode of public rental housing in China is mainly government construction and operation, which has the problems of low supply efficiency and low service level. It is critical to explore an efficient supply model in the construction of the GSRH system. Therefore, this study, starting from the three supply subjects of government, market, and society, constructs an evolutionary game model and uses agent-based modelling simulation to explore how multisubjects achieve optimal collaboration in the supply process of GSRH. The results are as follows: First, the development of a collaborative supply system includes four stages: noncooperative behaviour, collaborative exploration, collaborative game, and three-subject collaborative supply. Second, the government is the core of realising multisubject coordination. Increasing government supervision will boost market participation, while increasing government subsidies can fully mobilise the enthusiasm of social subjects but cannot continuously improve the market's enthusiasm. Third, increasing the participation ratio of social subjects will help mobilise the enthusiasm of other subjects to participate, while the excessive participation ratio of market subjects may cause an imbalance in the collaborative supply system. This study provides theoretical support for the efficient supply of GSRH.

## 1. Introduction

China is currently experiencing the largest scale of urbanisation ever known in human history, with its ratio expected to reach 69 percent in 2030 [1]. The seventh census results show that the national floating population was 376 million in 2020, an increase of nearly 70 percent in 10 years [2]. The acceleration of China's urbanisation process has led to strong demand for housing rentals among “new citizens” in the net inflow of population of big cities such as Beijing and Shanghai. The concept of “new citizens” refers to the population that flows from other places and lives stably in cities, including groups of migrant workers, newly employed college students, and other groups. Owing to the lack of housing affordability, the housing needs of this group are mainly concentrated in rental housing. However, for a long time, China's real estate market and housing security system have emphasised sales over rent, the housing rental market has not been fully developed, the structure of supply and demand is unbalanced, and the large-scale rental market has not been supported by special policies. Therefore, the new citizen group has become a “sandwich layer” group that neither is within the coverage of public rental housing (PRH) nor can afford commercial housing, bearing the housing pressure and crowding-out effect caused by high housing prices [3]; thus, it is a prominent problem that they cannot afford to buy a house or rent a good house at the present stage [4, 5]. To compensate for the obvious shortcomings of the housing security system and break the housing dilemma of the new citizens, in July 2021, the General Office of the State Council issued the “Opinions on Accelerating the Development of Government-subsidized Rental Housing (GSRH),” which clearly proposed the concept of GSRH in the top-level design of the housing security system [6]. However, the current policy only proposes the basic concept of GSRH, and scholars and policymakers still need in-depth discussions on supply subjects and supply models.

GSRH is an institutional innovation to alleviate the housing difficulties of new citizens [7]. At present, China has established a housing security system with PRH, GSRH, and coownership housing as the main body. Among them, PRH has two standard lines of low income and housing difficulties and is mainly aimed at urban low-income families in difficulty. The supply mode of PRH is directly led by the government and built on the platform of state-owned enterprises, and the government undertakes the responsibility of the bottom guarantee. GSRH is mainly used to solve the housing difficulties of the new citizen group to make up for the institutional innovation of China's previous imperfect housing security system for the same group. In terms of supply mode, GSRH reconstructs a market-oriented and policy-supported security system and guides multisubject investment and multichannel financing of “small-sized, low-rent” rental housing. GSRH is an important measure with Chinese characteristics that promotes the steady development of the real estate market and stabilises the position of “housing not for speculation.”

The single supply of the government subject has problems such as the obvious funding gap, the prominent contradiction between supply and demand, and weak construction enthusiasm caused by a one-sided view of political achievements. At present, the group of new citizens is large, and the shortage of rental housing is obvious. It is necessary for the housing security subject to change from government-based participation to the joint participation of the government, enterprises, and social forces to improve the efficiency and quality of supply. According to the policy background of GSRH, the supply subjects can be divided into government and nongovernmental institutions, in which the former refers to the public sector of local housing management institutions, while the latter are further divided into profit-oriented market subjects (including housing rental franchise enterprises, real estate development enterprises, and property service companies) and nonprofit social subjects (including large private enterprises, state-owned enterprises and institutions, industrial parks, research institutes, and rural collectives). The supply subject can be further abstracted as government, market, and society.

Therefore, this study focuses on how to cooperate with the government, market, and social subjects to participate in the rapid and effective supply of GSRH in large cities. Previous studies on PRH supply have mainly focused on four aspects as follows.

First, regarding the sustainability of PRH supply, [8] has enriched the knowledge of sustainable construction by proposing a building information modelling and life cycle assessment integration approach to comprehensively evaluate the life cycle-embodied environmental impacts of buildings at the design stage. Reference [2] constructs a sustainable development system of public housing project from the perspective of a complex eco-system, exploring the internal operation mechanism and the coupling mechanism among the ecological, economic, and social subsystems. Reference [9] assesses the financial viability of PRH projects in China from a private sector perspective. References [10, 11] propose the application of “green leasing” and “integrated design process” in low-cost housing. Reference [12] studies how to make affordable housing more sustainable from the perspective of stakeholders.

Regarding the relationship between central and local governments, some scholars have suggested that the central government delegates the responsibility of PRH supply to local governments, allowing these to obtain a set of administrative power in public engineering planning, maintenance, and commercial management [13]. Reference [14] believes that this decentralisation process shifts the burden of PRH supply from the state to local governments, with a view to creating new incentives at the microlevel to increase efficiency and productivity. Reference [15] studied the contradictions in China's PRH policy and found that the division of power, incentives, responsibilities, and income sources between the central and local governments runs counter to the national goal of affordable housing. Reference [16] researched campaign-style implementation and affordable housing provision in China, and the findings point to the defects of campaign-style implementation and China's need for more institutionalised mechanisms to implement policies prioritised by the national government.

With regard to local government supply problems, specific political and economic incentives for local governments played an important role in realising the scheme [17]. Many scholars have proposed that PRH brings negative effects such as a difficult integration into mainstream urban life, residential segregation, and job-housing balance [18, 19]. Reference [20] proposes that, driven by economic interests, local governments often construct high-capacity PRH communities in remote areas, thus potentially distorting the ambitious aims and principles of China's affordable housing scheme and negatively affecting the social interaction and life opportunities of the security objects. Reference [15] argues that, based on the interests of land, the spatial distribution of PRH development in China is biased towards the urban fringe to provide an institutional explanation. In view of China's intergovernmental relations, they believe that this discriminatory location approach is the result of urban government efforts to balance top-down political pressure and local fiscal interests.

Regarding changes in the supply mode of public housing, it is difficult for the government as a single subject to achieve the supply of PRH. Some scholars hold that the private sector has financial and professional advantages and can share risks with the public sector, reduce operating costs, introduce competition, increase options, and improve service quality [21, 22]. Using nongovernmental resources can create a more flexible structure to quickly adapt to rapidly changing environments [23]. Public-private partnerships (PPP) are widely seen as a way to involve the private sector with rich capital and management experience in sharing the financial burden of governments and improving the efficiency and sustainability of the public housing supply [24, 25]. In Austria, the UK, and Italy, innovative mixed arrangements for the development, financing, and management of indemnificatory housing jointly developed by the state, the third sector, market, and community participants show that they can benefit from the participation of market participants and communities [26]. Around 2011, the central government launched an initiative to encourage local governments to cooperate with nongovernmental organisations in providing public housing [27]. Reference [28] evaluated the feasibility of PPP in social housing. Reference [23] studied the structure and mechanism of the role played by nongovernmental actors in public housing governance.

Previous research on PRH supply has mainly focused on its sustainability, clarifying the relationship between central and local governments in the supply process as well as the problems existing in the supply of local governments and exploring the feasibility of public-private cooperation. However, a research gap exists with regard to how a collaborative government, market, and society can supply PRH. In the complex, self-adaptive system of multisubject supply of GSRH, the plan chosen by any subject of the government, market, and society affects the interests of other subjects, and the relationship between different subjects may lead to different supply results. Behaviour is also influenced, to some extent, by the surrounding environment [29, 30]; therefore, it is not enough to only observe the participants in the research on the multisubject supply of GSRH and, more importantly, the research on the relationship between the participants and the interaction with the system environment [31].

The supply of GSRH by the government, market, and society is a science about collaboration. German scholar Haken proposed the synergy concept in system theory, which meant a subsystem's collaboration, cooperation, synchronous combined effect, and collective behaviour in the system, that the whole realised the effect via nonlinearity complex interaction among the subsystem which the individual cannot realise to produce the 1 + 1 > 2 collaborative effect [32]. In the supply process, this synergy system is dynamic; that is, various factors influence each other through a relationship that is not only collaborative but also evolutionary [33]. Therefore, the supply of GSRH by government, market, and society is based on the coevolution theory. The coevolution theory follows the general analytical framework of Darwinism, which specifies the replicators and interactors in detail, and uses the “variation,” “replication,” and “selection” to describe the process of coevolution [34]. Evolutionary game theory (EGT) provides a useful method for Darwin's competition by defining a framework of competition, strategy, and analysis model [35].

EGT is based on the assumption of bounded rationality, taking the group as the research subjects, analysing its dynamic evolution process, and explaining why and how the group reaches the current evolutionary state [36, 37]. In the field of housing research, [38] built an evolutionary game model of the government and real estate operators in the housing rental market in the context of financial institutions and public participation in supervision and analysed the impact of different levels of supervision by financial institutions and the public on evolution. Reference [39] established an evolutionary game model between social forces and government to solve the problem of excessive participation of the former in housing rental projects leading to rising rents. Reference [40] constructed the interest game model between the central government and local governments in the process of developing rental housing, analysed the logic and dilemma of land reserve strategy and illegal land reserve problem, and determined the replication dynamic mechanism and evolutionary stability strategy of participants under various conditions. Therefore, this study explores the deep mechanism of supply system dynamic collaboration through a multisubject evolutionary game method based on coevolution theory.

In social systems, understanding a political or economic system requires more than an understanding of the individuals that comprise the system [41, 42]. It also requires understanding how the individuals interact with each other and how the results can be more than the sum of parts [43]. For this highly complex, nonlinear, and self-organising multiagent cooperative supply system based on an evolutionary game, agent-based modelling (ABM) can provide insights into dynamic interactions among real-world phenomena by capturing nonlinear interactions and feedback loops, thereby predicting outcomes that emerge out of complex dynamics in the real world [44–46]. ABM tools provide support for researchers and practitioners to study how the macrobehaviour of the system depends on the attributes, constraints, and rules at the microlevel and is increasingly recognised in ecology, economics, biology, sociology, social sciences, and many other STEM disciplines in simulating dynamic large-scale complicated systems and observing emergent behaviours [40, 47–49].

In summary, this study attempts to use EGT to explore the collaborative relationship of the three government-market-society subjects in the supply process, and it links the behaviour of agents with different information and decision rules with the macrobehaviour of the whole collaborative supply system with the help of the NetLogo multiagent simulation platform. Through large-scale policy experiments, it realises a bottom-up policy simulation of the game results and the collaborative supply mechanism of the three subjects; further, it explores the micromechanism of the evolution of the collaborative supply decision of GSRH under the condition of bounded rationality. The remainder of the paper is structured as follows.  Section 2 analyses the evolutionary game model and ABM of GSRH.  Section 3 establishes an asymmetric evolutionary game model of government, market, and society.  Section 4 establishes an agent-based model based on the EGT model through the NetLogo simulation platform and conducts large-scale policy experiments to determine the evolution stage and collaborative supply mechanism of the three subjects. This study provides theoretical support for the formulation of a coordinated supply policy of GSRH in the pilot cities.

## 2. Materials and Methods

### 2.1. Evolutionary Game Theory

This study adopts a dynamic replication evolutionary game method to establish a three-group, two-strategy, asymmetric evolutionary game model to study the dynamic process of government-market-society in the collaborative supply of GSRH.

#### 2.1.1. Game Agent Analysis

The supply subjects of GSRH can be abstracted into three categories: government, market, and society.

The government subject refers to the public sector of the local housing management institutions, including local government housing construction departments at all levels, housing security centres, and others. As quasipublic good, the supply of GSRH cannot and does not need to be fully borne by the government; however, as the executive department of state power, the government is fully responsible for guiding the supply of GSRH and has always been one of the main suppliers. Government participants rely on hierarchical guidance, mainly acting as nonprofit entities [23]. For the supply of GSRH, the state is at the top of the hierarchy, with the central government setting national supply policies and tasks and local governments acting as intermediaries responsible for establishing specific methods for local policy, project development and implementation, and negotiating with other actors to achieve housing construction and distribution [17, 50]. The authority and systematic organisation of government subjects can successfully promote the construction of GSRH, strengthen public services, ensure fair supply, and stabilise economic development. Moreover, the government subject is a key intermediary connecting other subjects [51].

The market subject refers to profit-making organisations that raise funds by themselves, including housing rental franchise enterprises, real estate development enterprises, property service companies, and others capable of directly or indirectly participating in the supply of GSRH. Market players use commercial principles to achieve efficiency, effectiveness, and innovation in providing public goods and social services [52]. The participation of market players reduces government costs and improves supply efficiency and supply effect. Moreover, it can avoid the psychological label effect of the centralised construction of GSRH on the security objects and achieve the goal of coordinating the layout of GSRH and ordinary commodity housing. The market subject is a key participant in effective supply; however, it is usually subject to the profit trend [19]. Compared with commercial projects, participation in GSRH projects has fewer returns and is not attractive to the market, and market subjects thus need certain incentives from the government. The incentive policies and measures are many, including reducing loan interest rates and providing tax relief and cheap or free land [2].

The social subject refers to nonprofit organisations that participate in supply on the principle of voluntary and mutual assistance. In China, these are large private enterprises, state-owned enterprises and institutions, industrial parks and rural collectives, and others. They build or reconstruct GSRH through self-construction, joint ventures, and shares or in other ways, using collectively operated construction land, idle land owned by enterprises and institutions, supporting land for industrial parks, and stock of idle houses. The social subject connects governments, collectives, and individuals and plays a complementary and balanced role in the multiagent collaborative supply system. Compared with government departments, social organisations are independent of the government system, which can get rid of the fixed pattern of the bureaucratic structure of government organisations and form their own unique diversity and flexibility, thus bringing low cost and high efficiency of supply. At the same time, it can effectively compensate for the lack of efficiency caused by the government's single supply or privatisation production mode. Compared with the private sector, the nonprofit goal of social organisations to participate in GSRH supply determines its more public welfare, the pursuit of high quality, and the efficiency of affordable rental housing supply.

#### 2.1.2. Model Hypothesis

Many factors affect the collaborative supply of GSRH by the government, market, and society. This study draws upon several relevant studies and empirical research works. To simplify the problem, the model is constructed and analysed based on satisfying the following assumptions.

**Assumption 1 .**

The increased delegation of government tasks to other nongovernmental actors has made the GSRH provision a “governance” model [26]. Therefore, the government has two strategic choices in the process of GSRH supply; the strategy space is {intervention, no intervention} ({I, NI}), in which intervention is divided into subsidies and supervision. Subsidy refers to the use of land policy support, fiscal and taxation relief, financial support, and other policy means by the government subject, guiding the market and social subjects through new reconstruction, idle transformation, and other modes to participate in the supply of GSRH [52]. Supervision means that the government should supervise and correct the relevant behaviours of the market subject and social subject in the planning, construction, and operation of GSRH, establish and improve the housing rental management service platform, strengthen the supervision of the whole process of construction, rental, and operation management of GSRH, and enhance the supervision of engineering quality and safety, especially the problems of unqualified construction quality and indoor decoration health. Nonintervention refers to the free development of the supply system of GSRH without formulating or implementing any subsidy and supervision policies.

**Assumption 2 .**

If the government adopts the intervention strategy, the cost of policy subsidies is *C*_*M*_ for the market subject and *C*_*S*_ for social players. Compared with the market subject, social players are more willing to participate in supply because the provision of GSRH is conducive to attracting talents and improving productivity. In addition, they generally maintain idle houses or land, and the construction costs are also small. Therefore, it is believed that *C*_*M*_ > *C*_*S*_. The supervision cost generated in the process of government intervention is *S*_*M*_ as the market subject and *S*_*S*_ as the social subject. Considering that the supply object of the social subject is unit workers, the supply quality directly affects the unit's operation efficiency through residential satisfaction, the self-discipline level of high-quality supply is high, and the difficulty and cost of government regulation are low; thus, it is assumed that *S*_*M*_ > *S*_*S*_.

**Assumption 3 .**

If the government takes a nonintervention strategy, local governments may obtain the leisure and governance resources, effective disposition, and so on, which bring extra gains to government subject as *R*_0_. Economic growth used to be and still is the main criterion for the central government to assess the promotion of local officials [53]; in other words, local officials are more willing to allocate land resources to infrastructure and real estate construction projects that generate significant fiscal revenues to improve promotion opportunities [54]. To realise virtuous interactions between the central government and local governments in the field of housing construction and management, it is proposed that the central government should change the single evaluation system for local officials and establish a multiangle and comprehensive performance evaluation system for local officials [55]. This study abstracts the central government's overall control of the supply of GSRH such that when the government subjects choose the nonintervention strategy, they are punished by the higher authorities as P.

**Assumption 4 .**

According to the social contract theory, in addition to the need to fulfil the economic contract, the market and social subjects are obliged to fulfil the social contract. Their behaviour must conform to society's expectations; they must do their duty for social and economic improvement and adjust to the changes in social environment to respond to the stakeholders' interests [33]. Therefore, the strategic space of market and social subject is set as {participation, nonparticipation} ({P, NP}). For market subjects, participation refers to the development and operation of affordable rental housing projects, and the supply target refers to the whole society meeting the population's guarantee conditions. “Nonparticipation” refers to not participating in the GSRH supply and instead investing in the construction of commercial housing and other real estate development projects. For social subjects, participation refers to the use of their own land or idle houses to build or rebuild leased houses nearby, and the supply objects are generally employees and families of the unit. “Nonparticipation” refers to not participating in the supply of GSRH and using investment within social organisations.

**Assumption 5 .**

For market players, adopting a participation strategy allows obtaining government subsidy *C*_*M*_ and direct rental housing operating income such as rent; value-added service charges are set as *R*_*M*0_, and indirect reputation gains such as corporate social responsibility are set as *R*_*M*1_. Investing in other projects, instead of participating in the supply of GSRH, yields *R*_*NM*_. For the social subjects, the government subsidy *C*_*S*_ is obtained by taking the participation strategy. The GSRH supplied by the social subjects mainly uses the stock of land and housing to build in the industrial park and around the company to effectively solve the problem of job-housing balance, improve the enthusiasm of employees, generate a positive income *R*_*S*0_, and bring a certain social reputation income *R*_*S*1_. Social reputation is the higher satisfaction of staff and the social influence of suppliers brought by the physical supply of rental housing. Given the nonprofitability of the social subjects supplying GSRH, the operating income of rental housing is not considered here. Not participating in the supply of GSRH and instead using other investments within social organisations enable receiving *R*_*NS*_, but for social subjects who do not participate, restraint measures shall be taken; that is, they need to pay a certain housing subsidy F to the unit's employees.

**Assumption 6 .**

GSRH belongs to the housing security system, which is essentially a certain degree of social welfare provided by the government. The main responsibility of it lies with the government, market, and social subjects through a collaborative supply. If market and social subjects participate in supply, government financial pressure is reduced, and the project's operation efficiency and quality, as well as are the government's reputation, are improved so that the contribution of the market subject participating in supply is *GB*_*M*_ and that of the social subject is *GB*_*S*_.

**Assumption 7 .**

**Figure 1:**
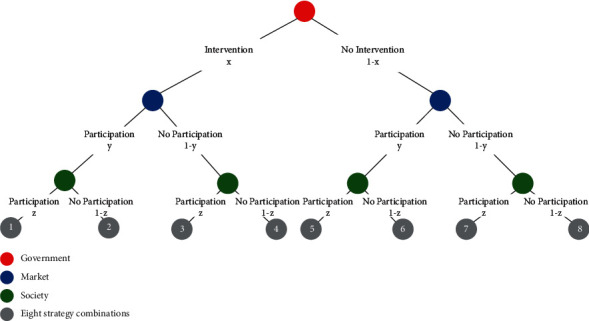
Dynamic flow of evolutional game among government, market, and social subject in GSRH supply.

Game participants are bounded rational. Without considering other constraints, government, market, and society all have the bounded rationality characteristic; in other words, each subject cannot accurately calculate its own cost and the income, but it usually tries and imitates unceasingly over time and eventually tends to a stable strategy. Suppose that the proportion of the government choosing the intervention strategy is *x*, the proportion of the market choosing the participation strategy is *y*, and the proportion of society choosing the participation strategy is *z* (0 ≤ *x*, *y*, *z* ≤ 1). The specific dynamic game flow of collaborative supply among government, market, and society is shown in Figure 1.

It is worth noting that the impact of the policy environment is complex, and no unified housing policy is applicable to the housing market of each city [56]. The supply of GSRH implements the strategy of “one city, one policy.” The key point of this strategy is to let the central government determine the basic principles and then allow local governments to determine the implementation details, which can make the real estate regulation of each city innovative and flexible and can, to a large extent, avoid the systemic risks arising from the unified regulation of the central government [55]. Therefore, this study does not make specific policy and institutional assumptions on the collaborative supply of government, market, and society, but it abstracts this supply as subsidies, benefits, and penalties to discuss the synergy system. The summary and description of all model parameters are shown in Table 1.

#### 2.1.3. Payoff Model

In the course of the evolutionary game, according to individual bounded rationality and limited information principle and based on the above model assumptions, the payoff matrix of the government-market-society asymmetric cooperative game is shown in Table 2.

According to the benefits of the strategy combination in Table 2, the expected revenue of the intervention strategy (*G*^(1)^), nonintervention strategy (*G*^(2)^), and average benefits ($\bar{U_{G}}$) adopted by the government subject is, respectively,

(1) $$\begin{array}{c} \left\{\begin{array}{c} U_{G^{\left(1\right)}}=yz\left(GB_{M}+GB_{S}-C_{M}-S_{M}-C_{S}-S_{S}\right)+y\left(1-\text{z}\right)\left(GB_{M}-C_{M}-S_{M}\;\right)\\ +\left(1-\text{y}\right)z\left(GB_{S}-C_{S}-S_{S}\right),\\ U_{G^{\left(2\right)}}=yz\left(GB_{M}+GB_{S}+R_{0}-\text{P}\right)+y\left(1-\text{z}\right)\left(GB_{M}+R_{0}-\text{P}\right)\\ +\left(1-\text{y}\right)z\left(GB_{S}+R_{0}-\text{P}\right)+\left(1-\text{y}\right)\left(1-\text{z}\right)\left(R_{0}-\text{P}\right),\\ \bar{U_{G}}=xU_{G^{\left(1\right)}}+\left(1-x\right)U_{G^{\left(2\right)}}. \end{array}\right. \end{array}$$

The replication dynamics equation of the government subject is therefore

(2) $$\begin{array}{c} F_{\left(\text{x}\right)}=\frac{{\text{d}}_{x}}{{\text{d}}_{t}}=\text{x}\left(U_{G^{\left(1\right)}}-\bar{U_{G}}\right)=\text{x}\left(1-\text{x}\right)\left[\text{P}-R_{0}-\text{y}\left(C_{M}+S_{M}\right)-\text{z}\left(C_{S}+S_{S}\right)\right], \end{array}$$

where *F(x)* indicates the rate of change in government intervention strategies, *F(x)* > 0 means that the government tends to adopt an intervention strategy, and *F(x)* < 0 means that the government tends to adopt a nonintervention strategy.

Similarly, the expected revenue of the participation strategy (*M*^(1)^), nonparticipation strategy (*M*^(*v*)^), and average benefits ($\bar{U_{M}}$) adopted by the market subject is, respectively,

(3) $$\begin{array}{c} \left\{\begin{array}{c} U_{M^{\left(1\right)}}=xz\left(C_{M}+R_{M}\right)+x\left(1-z\right)\left(C_{M}+R_{M}\right)+\left(1-x\right)zR_{M}+\left(1-x\right)\left(1-z\right)R_{M},\\ U_{M^{\left(2\right)}}=xzR_{N}+x\left(1-z\right)R_{N}+\left(1-x\right)zR_{N}+\left(1-x\right)\left(1-z\right)R_{N},\\ \bar{U_{M}}=yU_{M^{\left(1\right)}}+\left(1-y\right)U_{M^{\left(2\right)}}. \end{array}\right. \end{array}$$

The replication dynamics equation of the market subject is therefore

(4) $$\begin{array}{c} F_{\left(y\right)}=\frac{{\text{d}}_{\text{y}}}{{\text{d}}_{t}}=\text{y}\left(U_{M^{\left(1\right)}}-\bar{U_{M}}\right)=\text{y}\left(1-\text{y}\right)\left(xC_{M}+R_{M0}+R_{M1}-R_{NM}\right). \end{array}$$

The expected revenue of the participation strategy (*S*^(1)^), nonparticipation strategy (*S*^(2)^), and average benefits ($\bar{U_{S}}$) adopted by the social subject is, respectively,

(5) $$\begin{array}{c} \left\{\begin{array}{c} U_{S^{\left(1\right)}}=xy\left(C_{S}+R_{S}\right)+x\left(1-\text{y}\right)\left(C_{S}+R_{S}\right)+\left(1-x\right)yR_{S}+\left(1-x\right)\left(1-y\right)R_{S},\\ U_{S^{\left(2\right)}}=R_{NS}-F,\\ \bar{U_{S}}=zU_{S^{\left(1\right)}}+\left(1-z\right)U_{S^{\left(2\right)}}. \end{array}\right. \end{array}$$

The replication dynamics equation of the social subject is therefore

(6) $$\begin{array}{c} F_{\left(\text{z}\right)}=\frac{{\text{d}}_{\text{z}}}{{\text{d}}_{t}}=\text{z}\left(U_{S^{\left(1\right)}}-\bar{U_{S}}\right)=\text{z}\left(1-\text{z}\right)\left(xC_{S}+R_{S0}+R_{S1}-R_{NS}+F\right). \end{array}$$

Based on the nature of evolutionary stability strategy, the necessary condition for government subject to achieve evolutionary stability is d*F*(*x*)/d*x* < 0.

**Figure 2:**
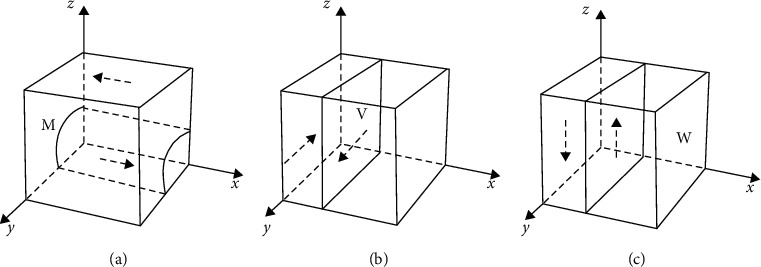
Replicated dynamic diagram of each subject. (a) Government. (b) Market. (c) Society.

For the replication dynamic equation (2), when *z*=(*P* − *R*_0_ − *y*(*C*_*M*_+*S*_*M*_))/(*C*_*S*_+*S*_*S*_), *x* is a steady state. When *z* < (*P* − *R*_0_ − *y*(*C*_*M*_+*S*_*M*_))/(*C*_*S*_+*S*_*S*_), *x*=1 is the evolutionarily stable strategy. When *z* > (*P* − *R*_0_ − *y*(*C*_*M*_+*S*_*M*_))/(*C*_*S*_+*S*_*S*_), *x*=0 is the evolutionarily stable strategy. According to *z*=(*P* − *R*_0_ − *y*(*C*_*M*_+*S*_*M*_))/(*C*_*S*_+*S*_*S*_), a surface *M* can be drawn, as shown in Figure 2(a). The points on surface *M* are stable in the *x*-axis direction, the points above it evolve to *x*=1, and the points below it evolve to *x*=0. Similarly, for the point of market subject in surface V to stabilise in the *y*-axis direction, the point on the left side of surface V evolves towards *y*=0, and the point on the right side of the surface tends to *y*=1, as shown in Figure 2(b). For the stability of the social subject in the *z*-axis direction of the point in plane W, the point on the left side of plane W evolves to *z*=0, and the point on the right side of it evolves to *z*=1, as shown in Figure 2(c).

### 2.2. Agent-Based Modelling

Based on the above evolutionary game model and ABM simulation method, with a bottom-up, this part simulates the evolution process of multiagent supply of GSRH under the collaborative background from the perspective of microinteraction to macroemergence, and it attempts to reveal the micromechanism of collaborative supply of GSRH in the form of policy experiments in view of the advantages of the NetLogo 6.0.3 simulation platform, which can meet the simultaneous operation of multiple agents, set system variables, and parameters easily and quickly and provide a visual evolution interface. With the help of this platform, this study establishes the attributes of each subject, the number of agents in each subject, the selection mechanism of game objects, and the strategy update mechanism, and it compiles the government-market-society, three-agent game simulation programme. Through NetLogo, large-scale numerical experiments are conducted to show the dynamic game process of different subjects under different parameter conditions, verify the evolutionary game model of multiagent synergistic supply of GSRH, realise the simulation and effect evaluation of relevant parameter and subject strategy selection changes, and provide verification for the effectiveness of policy recommendations.

#### 2.2.1. Basic Variable Settings of the NetLogo Simulation Platform

This study constructs a three-group, asymmetric evolutionary game model composed of agents and game environment and generates a two-dimensional network space with periodic boundary. In a two-dimensional network space, the three-party game subjects are generated as government, market, and social subjects, respectively.

The parameter settings of each subject attribute are shown in Table 3.

The strategy of the government subject is *S*_*G*_(t)={0,  1}, where 0 means adopting a nonintervention strategy, and 1 means adopting an intervention strategy. The strategy of the market subject is *S*_*M*_(t)={0,  1}, where 0 means adopting a nonparticipation strategy, and 1 means adopting a participation strategy. The strategy of the social subject is *S*_*S*_(t)={0,  1}, where 0 means adopting a nonparticipation strategy, and 1 means adopting a participation strategy.

The expected return functions of the three subjects of government, market, and society to adopt their respective strategies are as follows:

(7) $$\begin{array}{c} \left\{\begin{array}{c} IR_{G}\left(t\right)=y_{t}\left(GB_{M}-C_{M}-S_{M}\right)+z_{t}\left(GB_{S}-C_{S}-S_{S}\right),\\ NIR_{G}\left(t\right)=y_{t}GB_{M}+z_{t}GB_{S}+R_{0}-P,\\ PR_{M}\left(t\right)=x_{t}C_{M}+R_{M0}+R_{M1},\\ NPR_{M}\left(t\right)=R_{NM},\\ PR_{S}\left(t\right)=x_{t}C_{S}+R_{S0}+R_{S1},\\ NPR_{S}\left(t\right)=R_{NS}-F. \end{array}\right. \end{array}$$

#### 2.2.2. Simulation Mechanism Design

Agent game and movement mechanism: agents can move randomly in the network space, assuming that the game range of each agent in the interaction process is in a neighbouring position. Initially, each agent selects the corresponding strategy with a random probability. In the simulation period *t*, according to the agent's behaviour rules, the agent observes whether there are other agents around its eight neighbour positions. If there are no other agents, the agent moves to any neighbouring position randomly. If there are, it judges whether there are agents of other two types of subjects at the same time, and if this is the case, the game is randomly matched, and the strategy adopted at *t* + 1 is determined by learning the algorithm according to the benefits of the game.

Strategy learning mechanisms: since the agent is and asymmetrical information, it is not strictly based on the principle of maximum utility to make decisions in the game. According to the view of biological evolution and replication dynamics, the subject with lower returns continues to learn, imitate, and compare different strategies, then chooses a strategy higher than the expected return of this strategy, always replaces the unsatisfied strategy with a satisfied one, and finally tends to a stable state [38, 57]. The agent takes the result of each game as the actual benefit of its strategic interaction and then compares it with the expected benefit of the two strategies at the same time to judge whether the strategy of the *t*-period is optimal and whether to update it.

For the government subject, when the strategy of individual *i* in Agent_*G*_ at period *t* is *Str*_*G*(*i*)_(t)=1, if *NIR*_*G*(*i*)_(*t*) > *IR*_*G*(*i*)_(t) > *FR*_*G*(*i*)_(*t*) or *NIR*_*G*(*i*)_(*t*) > *FR*_*G*(*i*)_(*t*) > *IR*_*G*(*i*)_(t), then the strategy of Agent_*G*(*i*)_ at period *t* + 1 is *Str*_*G*(*i*)_(t+1)=0. When the strategy of individual *i* in Agent_*G*_ at period *t* is *Str*_*G*(*i*)_(t)=0, if *IR*_*G*(*i*)_(t) > *NIR*_*G*(*i*)_(*t*) > *FR*_*G*(*i*)_(*t*) or *IR*_*G*(*i*)_(t) > *FR*_*G*(*i*)_(*t*) > *NIR*_*G*(*i*)_(*t*), then the strategy of Agent_*G*(*i*)_ at period *t* + 1 is *Str*_*G*(*i*)_(t+1)=1, the other cases keep the original strategy unchanged, and the formula is as follows:

(8) $$\begin{array}{c} Str_{G\left(i\right)}\left(t+1\right)=\left\{\begin{array}{c} 1-Str_{G\left(i\right)}\left(t\right),\;Str_{G\left(i\right)}\left(t\right)=1\text{ and}NIR_{G\left(i\right)}\left(v\right)>IR_{G\left(i\right)}\left(t\right)>FR_{G\left(i\right)}\left(t\right)\\ \text{or}NIR_{G\left(i\right)}\left(t\right)>FR_{G\left(i\right)}\left(t\right)>IR_{G\left(i\right)}\left(t\right),\\ Str_{G\left(i\right)}\left(t\right)=0\text{ and}IR_{G\left(i\right)}\left(t\right)>NIR_{G\left(i\right)}\left(t\right)>FR_{G\left(i\right)}\left(t\right)\\ \text{or}IR_{G\left(i\right)}\left(t\right)>FR_{G\left(i\right)}\left(t\right)>NIR_{G\left(i\right)}\left(t\right),\\ Str_{G\left(i\right)}\left(t\right),\text{ othersituation}. \end{array}\right. \end{array}$$

Similarly, for the market subject, the strategy learning rules are as follows:

(9) $$\begin{array}{c} Str_{M\left(i\right)}\left(t+1\right)=\left\{\begin{array}{c} 1-Str_{M\left(j\right)}\left(t\right),Str_{M\left(j\right)}\left(t\right)=1\text{ and}NPR_{M\left(j\right)}\left(t\right)>PR_{M\left(j\right)}\left(t\right)>FR_{M\left(j\right)}\left(t\right)\\ \text{or}NPR_{M\left(j\right)}\left(t\right)>FR_{M\left(j\right)}\left(t\right)>PR_{M\left(j\right)}\left(t\right),\\ Str_{M\left(j\right)}\left(t\right)=0\text{ and}PR_{M\left(j\right)}\left(t\right)>NPR_{M\left(j\right)}\left(t\right)>FR_{M\left(j\right)}\left(t\right)\\ \text{or}PR_{M\left(j\right)}\left(v\right)>FR_{M\left(j\right)}\left(t\right)>NPR_{M\left(j\right)}\left(t\right),\\ Str_{M\left(j\right)}\left(t\right),\text{othersituation}. \end{array}\right. \end{array}$$

For the market, the strategy learning rules are as follows:

(10) $$\begin{array}{c} Str_{S\left(i\right)}\left(t+1\right)=\left\{\begin{array}{c} 1-Str_{S\left(k\right)}\left(t\right),Str_{S\left(k\right)}\left(t\right)=1\text{ and}NPR_{S\left(k\right)}\left(v\right)>PR_{S\left(k\right)}\left(t\right)>FR_{S\left(k\right)}\left(t\right)\\ \text{or}NPR_{S\left(k\right)}\left(t\right)>FR_{S\left(k\right)}\left(v\right)>PR_{S\left(k\right)}\left(t\right),\\ Str_{S\left(k\right)}\left(t\right)=0\text{ and}PR_{S\left(k\right)}\left(t\right)>NPR_{S\left(k\right)}\left(t\right)>FR_{S\left(k\right)}\left(t\right)\\ \text{or}PR_{S\left(k\right)}\left(t\right)>FR_{S\left(k\right)}\left(t\right)>NPR_{S\left(k\right)}\left(t\right),\\ Str_{S\left(k\right)}\left(t\right),\text{othersituation}. \end{array}\right. \end{array}$$

Evolutionary stable result output mechanism: the three types of agents go through a certain number of games in the simulation period (*t*) until the evolutionary equilibrium state of collaborative supply is finally reached. The output results of the final evolutionary stability of the government-market-society collaborative supply process are expressed as *x*(*t*), *y*(*t*), *z*(*t*) through the estimation of different strategy proportions of each game party. Based on the complexity of the real world, the NetLogo simulation model established in this study introduces the consideration of uncertainty to deepen the simulation results of the three-subject evolutionary game.

Assuming that the simulation results based on NetLogo have ±10% error compared with the real world, the error terms of the government, market, and social agents are set as *α*, *ß*, and *γ*, respectively, where (*α*, *β*, *γ*) ∈ (−0.1, 0.1) and *x*(*t*),  *y*(*t*),  *z*(*t*) are calculated as follows:

(11) $$\begin{array}{c} \left\{\begin{array}{c} x\left(t\right)=\frac{N_{S_{G}=1}t}{N_{G}}\times \left(0.9+\alpha \right),\\ y\left(t\right)=\frac{N_{S_{M}=1}t}{N_{M}}\times \left(0.9+\beta \right),\\ z\left(t\right)=\frac{N_{S_{S}=1}t}{N_{S}}\times \left(0.9+\gamma \right). \end{array}\right. \end{array}$$

## 3. Results

### 3.1. The Equilibrium Analysis of the Model

#### 3.1.1. Stability Analysis of the Evolutionary Game

By further solving the replicative dynamic equations composed of d*x*/d*t*=d*y*/d*t*=d*z*/d*t*= 0, 12 equilibrium points can be found in the cooperative game system of the government, market, and social subjects, which are *E*_1_ (0, 0, 0), *E*_2_(1, 0, 0), *E*_3_ (0, 1, 0), *E*_4_ (0, 0, 1), *E*_5_ (1, 1, 0), *E*_6_ (1, 0, 1), *E*_7_ (0, 1, 1), *E*_8_ (1, 1, 1), *E*_9_ ((*F*+*R*_*NS*_ − *R*_*S*0_ − *R*_*S*1_)/(*C*_*S*_), 1, (*C*_*M*_+*P* − *R*_0_ − *S*_*M*_)/(*C*_*S*_+*S*_*S*_)), *E*_10_ ((*R*_*M*0_ − *R*_*M*1_+*R*_*NM*_)/(*C*_*M*_), (*P* − *R*_0_)/(*C*_*M*_+*S*_*M*_),  0), *E*_11_ ((*F*+*R*_*NS*_ − *R*_*S*0_ − *R*_*S*1_)/(*C*_*S*_), 0, (*P* − *R*_0_)/(*C*_*S*_+*S*_*S*_)), *E*_12_ ((*R*_*M*0_ − *R*_*M*1_+*R*_*NM*_)/(*C*_*M*_), (*C*_*S*_+*P* − *R*_0_ − *S*_*S*_)/(*C*_*M*_+*S*_*M*_), 1). According to the above assumptions, all initial points and evolution points must satisfy 0 ≤ *x*,  *y*,  *z* ≤ 1 to have practical significance. The region surrounded by *E*_1_-*E*_8_ is the equilibrium solution of the evolutionary game. When condition 0 < (*F*+*R*_*NS*_ − *R*_*S*0_ − *R*_*S*1_)/(*C*_*S*_) < 1 is satisfied, it leads to (*F*+*R*_*NS*_ − *R*_*S*0_ − *R*_*S*1_)/(*C*_*S*_) > 1, abandoning *E*_9_ and *E*_11_. When condition *P* − *R*_0_ < 0 is satisfied, it leads to (*P* − *R*_0_)/(*C*_*M*_+*S*_*M*_) < 0, abandoning *E*_10_. When condition *C*_*M*_+*S*_*M*_+*C*_S_+*S*_*S*_ < *P* − *R*_0_ is satisfied, it leads to (*C*_*S*_+*P* − *R*_0_ − *S*_*S*_)/(*C*_*M*_+*S*_*M*_) > 1, abandoning *E*_12_. Therefore, the equilibrium point of the dynamic system is *E*_1_-*E*_8_, and these eight equilibrium points constitute the boundary of the evolutionary game domain. The stability of these equilibrium points in the evolutionary system can be obtained by the local stability analysis of the Jacobian matrix [58].

The Jacobian matrix of the evolutionary game system is as follows:

(12) $$\begin{array}{c} J=\left[\begin{array}{ccc} \frac{\text{d}x/\text{d}t}{\text{d}x} & \frac{\text{d}x/\text{d}t}{\text{d}y} & \frac{\text{d}x/\text{d}t}{\text{d}z}\\ \frac{\text{d}y/\text{d}t}{\text{d}x} & \frac{\text{d}y/\text{d}t}{\text{d}y} & \frac{\text{d}y/\text{d}t}{\text{d}z}\\ \frac{\text{d}z/\text{d}t}{\text{d}x} & \frac{\text{d}z/\text{d}t}{\text{d}y} & \frac{\text{d}z/\text{d}t}{\text{d}z} \end{array}\right],\\ =\left[\begin{array}{ccc} 1-2x\left[\text{P}-R_{0}-\text{y}\left(C_{M}+S_{M}\right)-\text{z}\left(C_{S}+S_{S}\right)\right] & -x\left(1-x\right)\left(C_{M}+S_{M}\right) & -x\left(1-x\right)\left(C_{S}+S_{S}\right)\\ y\left(1-y\right)C_{M} & \left(1-2y\right)\left(xC_{M}+R_{M0}+R_{M1}-R_{NM}\right) & 0\\ z\left(1-z\right)C_{S} & 0 & \left(1-2z\right)\left(xC_{S}+R_{S0}+R_{S1}-R_{NS}+\text{F}\right) \end{array}\right]. \end{array}$$

Table 4 shows the eigenvalues of the Jacobi matrix corresponding to each equilibrium point.

#### 3.1.2. Multiscenario Evolutionary Game Analysis

According to Lyapunov's stability theory, the asymptotic stability at the equilibrium point can be judged by the eigenvalues of the Jacobi matrix; that is, when all eigenvalues are negative, the equilibrium point is the stable point of the evolutionary game. It can be clearly judged from the eigenvalue results of the above equilibrium points that the stability of the synergic supply system of GSRH is affected by the value of the parameters; therefore, the evolutionary stable points of the system are discussed in eight cases as follows:

 
**Scenario 1:***P* − *R*_0_ < 0 and −*C*_*M*_ < *R*_*M*0_+*R*_*M*1_ − *R*_*NM*_ < 0 or *R*_*M*0_+*R*_*M*1_ − *R*_*NM*_ < −*C*_*M*_ and −*C*_*S*_ < *R*_*S*0_+*R*_*S*1_ − *R*_*NS*_+*F* < 0 or *R*_*S*0_+*R*_*S*1_ − *R*_*NS*_+*F* < −*C*_*S*_; that is, the additional benefit of the government subject in adopting a nonintervention strategy to transfer resources is greater than that of the higher-level punishment, and the benefit of the market and social subjects in adopting a participation strategy is less than that of the nonparticipation strategy regardless of whether there is an intervention subsidy of government subjects. Therefore, the government tends to adopt a nonintervention strategy, and the market and social subjects adopt a nonparticipation strategy. The corresponding strategy stability point is *E*_1_(0,0,0). In this case, the three subjects cannot form a cooperative relationship.
 
**Scenario 2:***P* − *R*_0_ < 0 and *R*_*M*0_+*R*_*M*1_ − *R*_*NM*_ < −*C*_*M*_ and  *R*_*S*0_+*R*_*S*1_ − *R*_*NS*_+*F* > 0; that is, the extra benefits of government subject transferring resources by adopting a nonintervention strategy are greater than the punishment of superior departments. When there is no government subsidy, the market subject's nonparticipation strategy benefits more than the participation strategy, while the social subject's participation in supply benefits more than nonparticipation. Therefore, the government tends to adopt the nonintervention strategy, the market adopts the nonparticipation strategy, and society adopts the participation strategy. The corresponding strategic stability point is *E*_4_(0,0,1).
 
**Scenario 3:***P* − *R*_0_ < 0 and *R*_*M*0_+*R*_*M*1_ − *R*_*NM*_ > 0 and −*C*_*S*_ < *R*_*S*0_+*R*_*S*1_ − *R*_*NS*_+*F* < 0 or *R*_*S*0_+*R*_*S*1_ − *R*_*NS*_+*F* < −*C*_*S*_; that is, the additional benefits of the transfer of resources by the government subject adopting the nonintervention strategy are greater than the penalties of the higher authorities, and without government subsidies, the benefits of the market subject adopting the participation strategy are greater than those of the nonparticipation strategy, while the benefits of the social subject participating are less than those of the subject not participating. Therefore, the government tends to adopt the nonintervention strategy, the market adopts the participation strategy, society adopts the nonparticipation strategy, and the corresponding strategy stability point is *E*_3_(0,1,0).
 
**Scenario 4:***C*_*M*_+*S*_*M*_ < *P* − *R*_0_ < *C*_*M*_+*S*_*M*_+*C*_*S*_+*S*_*S*_ or *P* − *R*_0_ < 0  and  *R*_*M*0_+*R*_*M*1_ − *R*_*NM*_ > 0 and *R*_*S*0_+*R*_*S*1_ − *R*_*NS*_+*F* > 0; that is, when the government subject chooses the nonintervention strategy, the punishment of the superior department is greater than the income, and the difference between punishment and income is greater than the cost paid by the intervention social subject and less than the cost paid by the intervention market and social subject. Alternatively, the benefits of nonintervention strategies adopted by government subjects are greater than the penalties imposed by higher authorities, and the benefits of participation in supply by market and social subjects are greater than those of nonparticipation in supply without government subsidies. Therefore, the government tends to adopt the nonintervention strategy, while market and social subjects adopt the participation strategy, and the corresponding strategy stability point is *E*_7_(0,1,1).
 
**Scenario 5:***C*_*M*_+*S*_*M*_ < *P* − *R*_0_ < *C*_*M*_+*S*_*M*_+*C*_*S*_+*S*_*S*_ or *C*_*M*_+*S*_*M*_+*C*_*S*_+*S*_*S*_ < *P* − *R*_0_ and *R*_*M*0_+*R*_*M*1_ − *R*_*NM*_ < −*C*_*M*_ and *R*_*S*0_+*R*_*S*1_ − *R*_*NS*_+*F* < −*C*_*S*_; that is, under the condition of government subsidies, the benefit of market and social subject participating in the supply is less than that of not-participating subjects, and if the government subject adopts the nonintervention strategy, the punishment of the higher authorities is greater than the benefit of resource transfer and utilisation, and the difference between punishment and benefit is greater than the cost of government intervention in the market and social subjects. Therefore, the government subject adopts the intervention strategy, while market and social subjects adopt a nonparticipation strategy, and the corresponding strategic stability point is *E*_2_(1,0,0).
 
**Scenario 6:***C*_*M*_+*S*_*M*_ < *P* − *R*_0_ < *C*_*M*_+*S*_*M*_+*C*_*S*_+*S*_*S*_ or *C*_*M*_+*S*_*M*_+*C*_*S*_+*S*_*S*_ < *P* − *R*_0_ and *R*_*M*0_+*R*_*M*1_ − *R*_*NM*_ > 0 or −*C*_*M*_ < *R*_*M*0_+*R*_*M*1_ − *R*_*NM*_ < 0 and *R*_*S*0_+*R*_*S*1_ − *R*_*NS*_+*F* < −*C*_*S*_; that is, the benefits of nonparticipation in supply are greater than participation, and the social subject tends to adopt a nonparticipation strategy. When the government subject adopts a nonintervention strategy, the loss is greater than the cost of intervening in the market subject. Alternatively, the government subject adopts an intervention strategy, and the benefit of a market subject participating in supply is greater than that of nonparticipation. The government tends to adopt the intervention strategy, and the market tends to adopt the participation strategy. Thus, the corresponding policy stability point in this scenario is *E*_5_(1,1,0).
 
**Scenario 7:***C*_*M*_+*S*_*M*_ < *P* − *R*_0_ < *C*_*M*_+*S*_*M*_+*C*_*S*_+*S*_*S*_ or *C*_*M*_+*S*_*M*_+*C*_*S*_+*S*_*S*_ < *P* − *R*_0_ and *R*_*M*0_+*R*_*M*1_ − *R*_*NM*_ < −*C*_*M*_ and *R*_*S*0_+*R*_*S*1_ − *R*_*NS*_+*F* > 0 or −*C*_*S*_ < *R*_*S*0_+*R*_*S*1_ − *R*_*NS*_+*F* < 0; that is, the market subject tends to adopt a nonparticipation strategy when it benefits more from nonparticipation in supply than participation, when the loss of government subject without intervention is greater than the cost of interfering with the social subject and when the benefit of social subject participating in supply is greater than that of nonparticipation. When the benefit of social subject participating in supply is greater than that of nonparticipation under the intervention of the government subject, the government tends to adopt the intervention strategy, and society adopts the participation strategy. Thus, the corresponding policy stability point in this scenario is *E*_6_(1,0,1).
 
**Scenario 8:***C*_*M*_+*S*_*M*_+*C*_*S*_+*S*_*S*_ < *P* − *R*_0_  and *R*_*M*0_+*R*_*M*1_ − *R*_*NM*_ > 0 or −*C*_*M*_ < *R*_*M*0_+*R*_*M*1_ − *R*_*NM*_ < 0 and *R*_*S*0_+*R*_*S*1_ − *R*_*NS*_+*F* > 0 or −*C*_*S*_ < *R*_*S*0_+*R*_*S*1_ − *R*_*NS*_+*F* < 0; that is, when the loss of the government adopting a nonintervention strategy is greater than the cost of adopting an intervention strategy, the government subject must tend to adopt an intervention strategy. Under the intervention of the government subject, the benefit of the market and social subjects participating in supply is greater than that of not participating; thus, these subjects tend to adopt the participation strategy. Therefore, the corresponding strategy stability point in this scenario is *E*_8_(1,1,1).

In conclusion, the collaborative supply of GSRH can be divided into five stages: noncooperative behaviour, invalid exploration stage, collaborative exploration, collaborative game, and three-subject collaborative supply. Among them, Scenario 1 corresponds to the stage of noncooperative behaviour, in which the central government has a low level of control over the supply of GSRH, and local governments are more inclined to transfer investment to other projects that can generate considerable fiscal revenue. In Scenarios 2–4, the central government's control is still insufficient, local governments still choose a nonintervention strategy, and the benefits of participation by the market or social subject are greater than nonparticipation. Based on the quasipublic goods properties of GSRH, the government subject is the core of the GSRH supply, and the market and social subject cannot actively participate in it without the government's guidance; therefore, Scenarios 2–4 represent an invalid exploration stage. As the central government further intensifies its control over the GSRH supply, the government subject begins to choose the intervention strategy; thus, the supply system enters the collaborative exploration stage, corresponding to Scenario 5. In the process of exploring the intervention strategy of the government subject, intervention strategies are constantly adjusted to attract social or market subject to participate in supply, and the collaborative system enters the collaborative game stage, corresponding to Scenarios 6-7. In the collaborative game among government, market, and society, the three subjects constantly adjust their strategy choices and finally reach the collaborative supply stage of government intervention, market, and social participation, corresponding to Scenario 8. Given that GSRH is a kind of social welfare housing without government policy support and guidance, other subjects do not take the initiative to participate in the driving force. Therefore, the invalid exploration stage without the participation of the government subject is only a theoretical derivation result, which is not in line with the realistic logic of affordable rental housing supply and will not be discussed in subsequent research.

### 3.2. Simulation Analysis

This section explores the characteristics of various mechanisms and a more effective mechanism configuration through scenario simulation [59]. Based on the aforementioned setting of government, market, and society, the number of government, market, and social subjects is approximated by county administrative area, real estate enterprises, state-owned enterprises, and institutions, respectively. According to the statistical yearbook, in 2019, there were 2,792 county-level administrative regions, 94,790 real estate enterprises (except state-owned and collective investment enterprises), and 20,486 state-owned enterprises and institutions (in all industries) [60]. It can be concluded that the proportion of government, market, and social agents can be roughly estimated as 1 : 34 : 7. In this proportion, it is assumed that the initial state of the system is Agent_*G*_=10,  Agent_*M*_=340,  Agent_*S*_=70, and the initial strategy selection proportion of government, market, and social agents is 50%. On the NetLogo simulation platform, with a *t* = 500 simulation period and the probability of positive strategy selection of three game subjects as the main measurement standard, 50 policy simulation numerical experiments are conducted on the simulation parameters of each group. The following simulation results are obtained by analysing and visualising the experimental data.

#### 3.2.1. Experiment 1: Simulation Results and Analysis of the Evolutionary Game of the Three Subjects of Government-Market-Society

According to the above EGT derivation results, this section verifies the stability of the evolutionary equilibrium state of the GSRH collaborative supply system in each stage. The invalid exploration stage of EGT is only a theoretical derivation result that does not conform to the realistic logic of public housing supply in China. Therefore, this part only conducts a simulation analysis and discussion on the noncooperative behaviour stage, collaborative exploration stage, collaborative game stage, and collaborative supply stage. The parameter settings of each scenario are *P*_*S*_=(*GB*_*M*_, *GB*_*S*_, *C*_*M*_, *C*_*S*_, *S*_*M*_, *S*_*S*_, *R*_*M*0_, *R*_*M*1_, *R*_*NM*_, *R*_*S*0_, *R*_*S*1_, *R*_*NS*_, *R*_0_, P, F), and the corresponding scenarios in each evolution stage are shown in Table 5.

**Figure 3:**
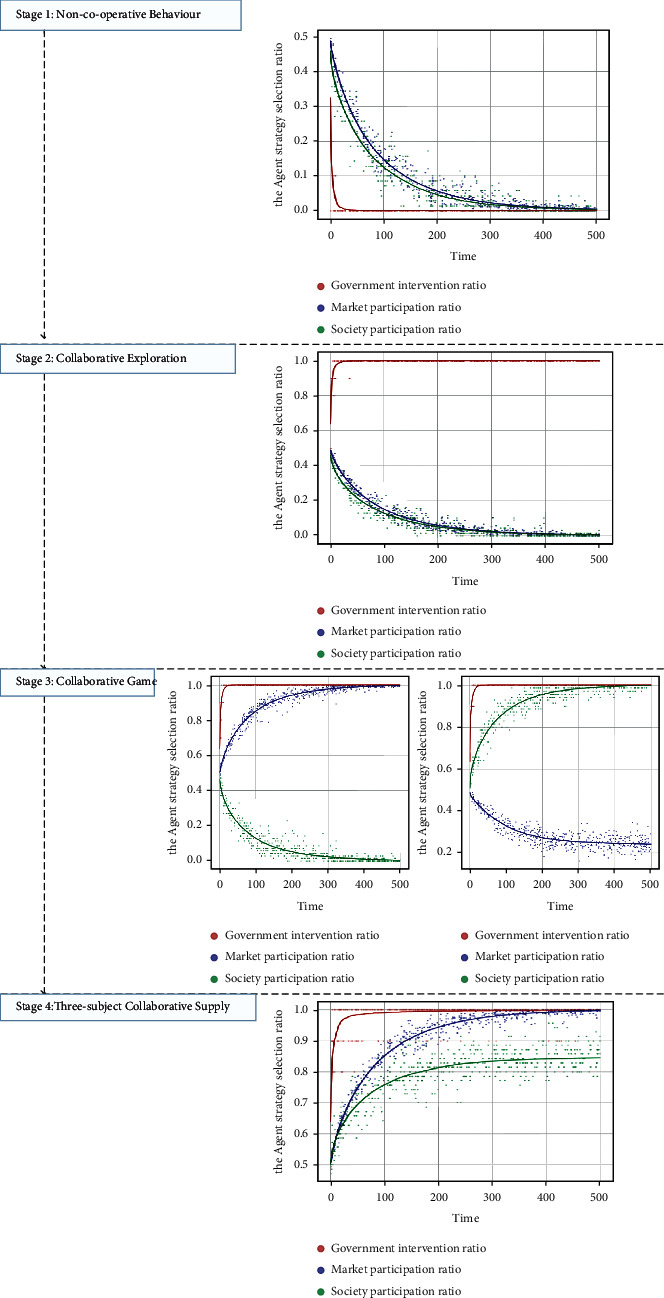
Four-stage simulation results of “government-market-society,” three-subject collaborative supply. (a) Stability test of equilibrium point (0, 0, 0). (b) Stability test of equilibrium point (1, 0, 0). (c) Stability test of equilibrium point (1, 1, 0). (d) Stability test of equilibrium point (1, 0, 1). (e) Stability test of equilibrium point (1, 1, 1).

When the government chooses the intervention strategy with a probability of 0.5 and the market and society choose the participation strategy with a probability of 0.5, the initial state of the evolutionary game system is (0.5, 0.5, 0.5). Throughout these four stages, in the stage of noncooperative behaviour, the punishment of the government subject for nonintervention by the higher authorities is less than the income, and the evolution result is that all the government agents tend to adopt the nonintervention strategy at a fast speed, and the final evolution is stable for the government subject to choose the intervention strategy with a probability of 0. With the decrease in the proportion of government agents taking intervention strategies, the probability of market and social agents taking that strategy tends to 0 at an approximate rate, as shown in Figure 3(a). When the central government pays more attention to the supply of GSRH and takes it as an important indicator for the performance evaluation or urban development of the government subject, the government subject tends to adopt the intervention strategy at a high rate, and the supply system of the city enters the stage of collaborative exploration. As the government subject has bounded rationality and obtains incomplete information in this phase, the enthusiasm of the market and social subjects to participate in the supply is not aroused; therefore, they will tend to adopt the nonparticipation strategy with a probability of 1 at a similar rate, as shown in Figure 3(b). Under the Further increase of subsidies and appropriate intervention by government subject on the market or society subject will attract the participation of the market or social subject and enter the cooperative game of the multisubject supply of GSRH, as shown in Figures 3(c) and 3(d)). In this stage, government agents need to adjust their strategy, actively intervene against nonparticipating agents, and conduct appropriate subsidies and supervision for active participation. In this process, each agent continues to learn and update its strategy; the intervention of the government subject successfully mobilises the enthusiasm of the market and social agents for participating, achieving the dynamic and stable state of the three-subject collaborative supply, as shown in Figure 3(e).

Note: in each evolution stage, the output results of the stability test of each equilibrium point are set as the average values of several parameters in the corresponding scenario.

#### 3.2.2. Experiment 2: The Impact of Government Intervention on Supply Efficiency in the Collaborative Supply Phase

In the collaborative supply stage of GSRH, different levels of government intervention may have different impacts on the efficiency of collaborative supply, in accordance with the hypothesis that the government's intervention strategy involves subsidies and supervision. To determine the impact of government intervention on supply efficiency, this section designs the following two sets of policy experiments.

The first experiment: for the supervision strategy, the government should increase or relax supervision to give full play to the market advantage to improve supply efficiency. Previous studies have asserted that strengthening government control can promote greater cooperation among various subjects in public housing supply [18], and the efficiency of public housing supply is a constraint of the government [17]; however, some scholars believe that it is necessary to provide the space for the market to play its advantage.

**Figure 4:**
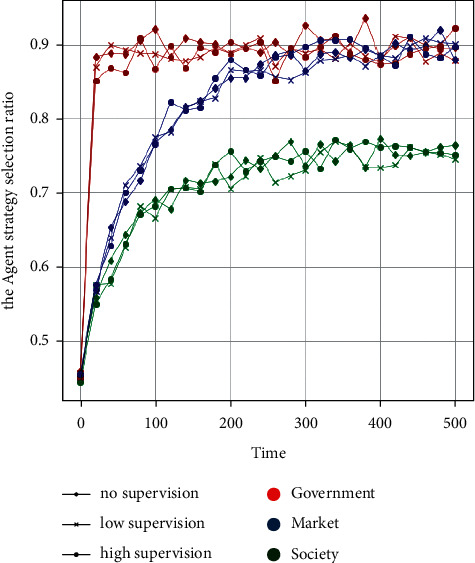
The influence of government supervision on the strategy selection of each agent.

To clarify this problem, this part controls the subsidy strategy and conducts no supervision, low-supervision, and high-supervision as three groups of parameters, respectively, to conduct the policy experiment on the influence of supervision intensity on the supply system. With *t* = 500 as the cycle, 50 policy simulations are conducted. The parameter settings of the no supervision, low-supervision, and high-supervision strategies are *P*_*S*1−0_=(5,5,1.5, 1,0,0,3,2,4,2,2,5,1,9,2), *P*_*S*1−1_=(5,5,1.5, 1,1.5, 1,3,2,4,2,2,5,1,9,2), and *P*_*S*1−2_=(5,5,1.5, 1,3,2,3,2,4,2,2,5,1,9,2).  Figure 4 shows the data analysis and visualisation of the output results of large-scale numerical experiments.

From the output results of the above large-scale policy simulation experiments, compared with the low-supervision strategy, when the government agents adopt the no supervision strategy, they do not have to pay the supervision cost, and the cost of participating in the supply is significantly reduced. Therefore, the proportion of government agents choosing the intervention strategy increases significantly during the simulation cycle. Compared with the low-supervision strategy, when the supervision is further increased, the proportion of government entities choosing intervention strategies decreases significantly as the intervention cost increases, and it is lower than the low-supervision strategy. In the early stage of three-subject collaborative supply (*T* < 100), no supervision, low-supervision, and high-supervision strategies have no significant effect on the strategy choice of market agents, and the market participation rate of the low-supervision strategy is higher relatively. With the development of the supply system (100 < *T* < 400), the participation rate of market agents choosing a high-supervision strategy is the highest, while the no supervision and low-supervision strategies have no significant impact on the market participation rate. Conversely, as the collaborative supply system matures (*T* > 400), “high regulation” restrains the participation rate of market agents.

For the market agents, the simulation results show that, in the early stage of the GSRH collaborative supply, the government's adoption of the low-supervision strategy is relatively efficient and plays a market advantage while regulating the market. In the development stage of the collaborative supply, the government may choose the high-supervision strategy to strengthen its control over the market and promote greater cooperation between the government and market agents [17]. The simulation results also show a significant increase in market participation under the high-supervision strategy. In the mature stage of the collaborative system development, high-supervision restricts the market, while a low-supervision strategy is more efficient. For social agents, the influence of government supervision on their participation rate is not obvious, indicating that the choice of a government supervision strategy is not a key factor for social agents to participate in the supply of GSRH.

**Figure 5:**
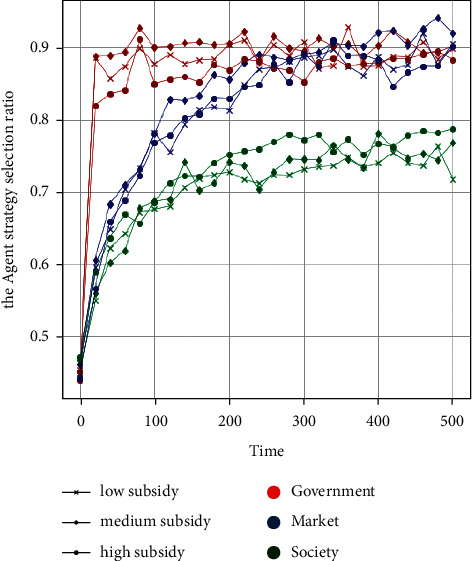
The influence of government subsidy on strategic selection of each agent.

The second experiment: how much policy subsidy can realise the efficient synergy of GSRH supply by the government subject with the market and social subjects? To clarify this problem, three strategies of low subsidy, medium subsidy, and high subsidy are set up to conduct policy experiments on the effect of subsidy degree on the supply efficiency of affordable rental housing under the control of supervision. With *t* = 500 as the cycle, 50 policy simulations are conducted. The parameter settings of the “low, medium, and high” strategy are *P*_*S*2−0_=(5,5,1.5, 1,1.5, 1,3,2,4,2,2,5,1,9,2),  *P*_*S*2−1_=(5,5,2,1.5, 1.5, 1,3,2,4,2,2,5,1,9,2), and *P*_*S*2−2_(5,5,3,2,1.5, 1,3,2,4,2,2,5,1,9), 2), respectively.  Figure 5 shows the data analysis and visualisation of the output results of large-scale numerical experiments.

It can be seen from the output results of the large-scale policy simulation experiment that a medium-subsidy strategy can mobilise the enthusiasm of various agents to participate compared with the low-subsidy strategy for government agents and the participation rate of government agents increases accordingly. While adopting the high-subsidy strategy, the government's main body is involved in the supply of financial burden overweight, leading to a reduction in the participation rate of government agents. The medium-subsidy strategy has brought a greater guarantee to the income of the supply of affordable rental housing for market players compared with the low-subsidy strategy, and the market participation rate has significantly increased. The market participation rate is lower when the high-subsidy strategy, rather than the medium-subsidy strategy, is adopted, indicating that the government's blind increase of subsidies for the market subject cannot continuously stimulate the enthusiasm of market participation and that this increase should be reasonable. As the social subject participates in the nonprofit supply of GSRH, it is more motivated with a “high subsidy” than with a medium or low subsidy.

#### 3.2.3. Experiment 3: Effects of Market and Social Participation on System Equilibrium

For different development stages of the collaborative GSRH supply system, the different initial strategy choices of the market and social agents may have different degrees of impact on the development of each stage of the collaborative supply system. To clarify this issue, this section designs the following two sets of experiments.

**Figure 6:**
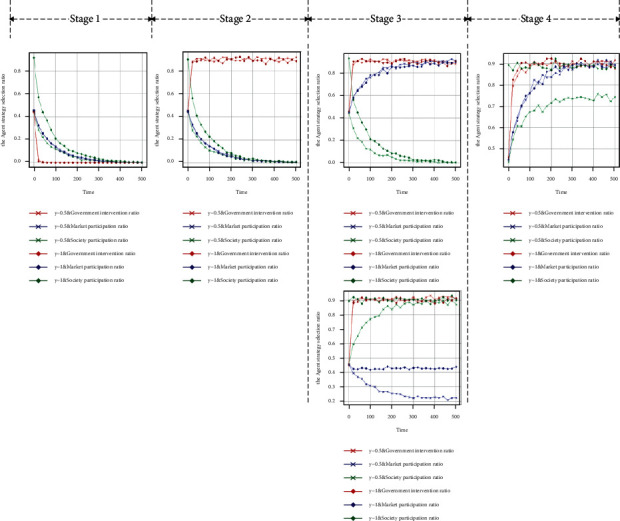
The influence of initial proportion of market agents' participation strategy on the supply system. (a) *y* = 0.5, *y* = 1, and stability test of equilibrium point (0, 0, 0). (b) *y* = 0.5, *y* = 1, and stability test of equilibrium point (1, 0, 0). (c) *y* = 0.5, *y* = 1, and stability test of equilibrium point (1, 1, 0). (d) *y* = 0.5, *y* = 1, and stability test of equilibrium point (1, 0, 1). (e) *y* = 0.5, *y* = 1, and stability test of equilibrium point (1, 1, 1).

The first experiment: two groups of strategies of *y*=0.5 and *y*=1 are set to conduct the policy experiment on the influence of the initial proportion of market agents in the participation strategy on the GSRH supply efficiency. With *t* = 500 as the cycle, 50 policy simulations are conducted.  Figure 6 shows the data analysis and visualisation of the output results of large-scale numerical experiments.

At every stage, when the probability of market participation is 1, compared with 0.5, the intervention rate of government agents is significantly reduced as the simulation results indicate that the participation rate of market agents increases in the collaborative supply stage (Stage 4). The participation rate of social agents is also greatly reduced at the same time, while there is no significant influence on the strategy choice of each subject in other stages, as shown in Figure 6(e). The simulation results show that the enthusiasm of market players is excessively aroused in the stage of collaborative supply, and the proportion of market agents occupy an excessively high proportion of the supply system, which leads to the relaxation of government intervention in supply and to social agents being squeezed out of the supply system by the market, resulting in the imbalance of the government-market-society collaborative supply system. The supply of GSRH is vulnerable to “market failure”; therefore, the government should take the initiative to regulate the participation ratio of market agents to avoid market-led supply imbalance in Stage 4.

**Figure 7:**
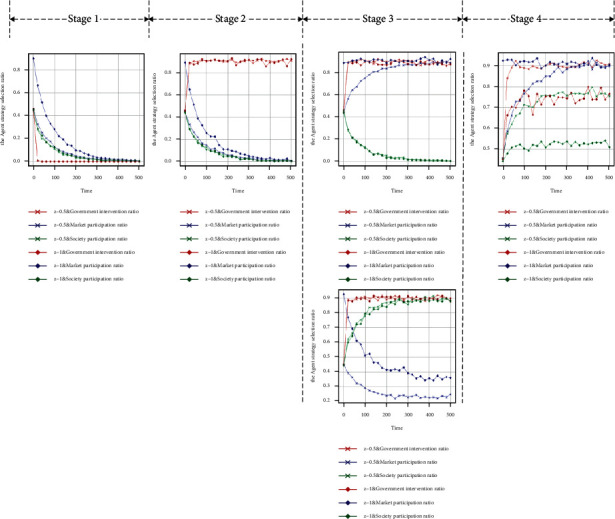
The influence of initial proportion of social agents' participation strategy on supply system. (a) *z*=0.5, *z*=1, and stability test of equilibrium point (0, 0, 0). (b) *z*=0.5, *z*=1, and stability test of equilibrium point (1, 0, 0). (c) *z*=0.5, *z*=1, and stability test of equilibrium point (1, 1, 0). (d) *z*=0.5, *z*=1, and stability test of equilibrium point (1, 0, 1). (e) *z*=0.5, *z*=1, and stability test of equilibrium point (1, 1, 1).

The second experiment: two groups of strategies of *z*=0.5 and *z*=1 are set to conduct the policy experiment on the influence of the initial proportion of market agents in the participation strategy on the GSRH supply efficiency. With *t* = 500 as the cycle, 50 policy simulations are conducted.  Figure 7 shows the data analysis and visualisation of the output results of large-scale numerical experiments.

At every stage, the probability of social participation is 1, compared with 0.5 when the evolutionary stability strategy is (1, 0, 1), namely, government intervention, market participation, and social participation, and the participation rate of market agents significantly improves with the increase of the participation rate of social agents in the collaborative game stage (Stage 3) from the simulation results, as shown in Figure 7(d). In the collaborative supply stage (Stage 4), as the participation rate of social agents increases, the market participation rate fluctuates, but the change is not significant, while the government intervention rate clearly increases, as shown in Figure 7(e). The impact on the strategy choice of each agent in other stages is not significant. The simulation results show that the increase of the proportion of social participation helps to mobilise the enthusiasm of market participants in the collaborative game stage and the government agents to intervene in the collaborative supply stage. Therefore, in the process of collaborative supply system development, mobilising the participation enthusiasm of social agents is conducive to the positive development of the collaborative supply system.

## 4. Discussion

On the basis of confirming previous studies, our results have obtained some new and more specific conclusions. First, the collaborative supply of GSRH can be divided into four stages: (i) noncooperative behaviour, (ii) collaborative exploration, (iii) collaborative game, and (iv) three-subject collaborative supply. Our division of the development stages of the multisubject collaborative supply system fills the gap of systematic research on the multisubject collaborative supply of public housing. The policy simulation of the four stages shows that the government is at the core of realising a multisubject collaborative supply, and the solution to housing affordability cannot rely solely on market forces. This result confirms the previous research conclusion on the important role of government subjects in the supply of affordable housing [26]. In the context of neoliberalism, Australia's study of the housing crises in Sydney and Melbourne also suggests that local governments must play a bigger role [61]. The policy's experimental findings further confirm the important role of specific political and economic incentives for local governments in achieving programmes [17]. The government should provide appropriate guidance for the market and social subjects, and it should be cautious in the intervention process. Improper intervention may create an uncontrollable and uncertain environment that affects market efficiency and fairness [62, 63]. And the government should determine the corresponding intervention measures according to the matching of supply and demand of GSRH in each city and the development stage of collaborative supply system so as to maximise the social benefits of these incentive mechanisms and ensure the financial feasibility of the market and social subjects [64].

Second, as the link for realising market and social synergy, how the government can efficiently realise the supply of GSRH is critical to the sustainable development of public housing at the stage of collaborative supply. The government's supervision and guidance strategy on the market and social subjects is equivalent to the “carrot” and “stick” initiative. A previous study discussed the feasibility of introducing housing affordability contributions and incentives when developers entered the planning process and pointed out that optimum scenarios identified a balance of carrots and sticks [64]. Based on the previous related research, combined with the development stage of the collaborative GSRH supply system, this study determines the government's supervision and guidance of market and social subjects in different development stages of the supply system and obtains more detailed results. The incentive measures applicable to the supply of GSRH in all cities do not exist. Therefore, in the process of realising the multisubject collaborative supply of public housing, the development stage of the collaborative supply system should be determined according to the basic situation of the city, such as the specific population, economic situation, and policy background, combined with the exploration of the multisubject collaborative model of the city, such as public-private partnerships and inclusionary housing. And then, the specific supply policy applicable to the city should be determined according to the development stage of the collaborative supply system and the national support policy.

Finally, affordable housing is increasingly developed, financed, and managed by a mix of state, third sector, market, and community actors. Previous studies on public-private cooperation have focused more on the significance of cooperation among government and private and nonprofit organisations in increasing the supply of indemnificatory housing [26, 65], and private real estate developers have entered into partnerships with the Housing Authority to finance, design, build, and manage the new developments [66, 67]. Based on previous studies, through large-scale policy simulation experiments, this study draws microscopic and more instructive conclusions and discusses the positive and negative effects of different participation ratios of the market and social subjects in the multisubject, collaborative supply. For the different development stages of the collaborative GSRH supply system, the different initial strategy choices of the market and social agents have different degrees of influence on the development of each stage. In different stages, reasonable control of the proportion of the market and social subjects choosing participation strategies is conducive to the balance of the collaborative supply system and to avoid an imbalance caused by the insufficient or excessive participation enthusiasm of market and social subjects.

## 5. Conclusion

In large cities with a net population inflow, the supply of GSRH is an important measure to alleviate the staged housing difficulties of new citizens and young people, and it plays an important role in promoting new urbanisation and optimising the spatial allocation of human capital in China. The coordination degree of government, market, and society in the supply process directly affects the implementation of the urban GSRH supply plan. Based on the multisubject evolutionary game model, this study establishes an agent-based model with the help of the NetLogo simulation platform, conducts large-scale policy experiments to explore the development status of the collaborative supply system of GSRH and the influence of each agent's strategic choices on the balance of the supply system, and draws the following conclusions.

First, the collaborative supply of GSRH can be divided into four stages: (i) noncooperative behaviour, (ii) collaborative exploration, (iii) collaborative game, and (iv) three-subject collaborative supply. Second, this study determines the government's supervision and guidance of market and social subjects in different development stages of the supply system and obtains more detailed results. (a) Market agents are sensitive to the government's choice of supervision strategy. The government should adopt different levels of supervision strategies at different stages of the development of the collaborative supply system (Stages I–II: supervision; Stage III: increased supervision; Stage IV: supervision), but government supervision is not a key factor affecting the participation of social subjects in supply. Therefore, the government can appropriately increase the supervision of the market and relax the supervision of social subjects in the process of coordination. (b) For the choice of subsidy strategy, the reaction of market agents to the intensity of the government's subsidy strategy has certain limits, beyond which the increase of government subsidies cannot mobilise the market's enthusiasm, while increasing subsidies to social agents can fully mobilise their participation enthusiasm. Therefore, the government should appropriately increase subsidies to market subject and give full subsidies to social subject. Finally, the results of the policy simulation experiment indicate that increasing the participation ratio of social agents helps mobilise the enthusiasm of the government and of market agents to participate in Stage III. However, the excessive participation ratio of market agents leads to the imbalance of the collaborative supply system in Stage IV; thus, the government subject needs to limit the market participation ratio within a reasonable range.

This study has strong theoretical and practical implications for the establishment of collaborative supply system of various public housing. At the theoretical level, in view of the dilemma of the GSRH supply, this study attempts to explore the internal mechanism of the multisubject collaborative supply of GSRH based on the synergy theory and the methods of EGT and ABM. The study enriches the research achievements related to collaborative supply and provides a certain scientific basis for the pilot cities to formulate specific supply policies. In terms of practical implications, this study first clarifies the core status of government subject in the supply of GSRH and provides guidance for the intervention of government subject. Secondly, A “city-specific policies” approach must be followed to avoid distortion of policy results and improper allocation of government resources so as to achieve rapid and efficient GSRH supply [14]. Finally, this study is of great significance to protect the right of residence in human development opportunities.

This study suffers from some limitations that provide directions for future research. First, the supply of GSRH in each pilot city is currently in the planning stage; therefore, the simulation model in this study lacks testing of actual cases. Secondly, the policy simulation experiment lacks quantitative results, and there is no clear quantification of the degree of supervision and guidance strategies that government subjects should give to other supply subjects in different development stages of the collaborative supply system. Moreover, there is no clear quantification of the reasonable participation ratio of market and social subjects in each development stage of this system. Future research will focus on the pilot cities of GSRH, bringing the empirical analysis data into the policy simulation model, quantifying the model, and enriching the existing research conclusions. Further, it will make this research model a policy decision-making tool based on the background research on the pilot city's development, input the research results into the model, and then provide policy suggestions with direct guiding significance for the supply of GSRH in the city.

## Figures and Tables

**Table 1:** Evolutionary game model parameters and variable descriptions.

Parameter	Description
*GB*_*M*_	Benefits of market subject's participation in supply to government subject
*GB*_*S*_	Benefits of social subject's participation in supply to government subject
*C*_*M*_	The cost of policy subsidies for market subject by government intervention strategies
*C*_*S*_	The cost of policy subsidies for social subject by government intervention strategies
*S*_*M*_	Supervision cost of government intervention strategy for market subjects
*S*_*S*_	Supervision cost of government intervention strategy for social subjects
*R*_*M*0_	Direct operating income from market participation strategy
*R*_*M*1_	Indirect reputation gains from market participation strategy
*R*_*NM*_	Returns from market nonparticipation strategy to investment in other projects
*R*_*S*0_	Positive benefits of social participation strategy
*R*_*S*1_	Reputation gains of social participation strategy
*R*_*NS*_	Benefits from social nonparticipation strategies to other projects in investment organisations
*R*_0_	Government gains from the additional benefits of leisure and the efficient allocation of government resources when it takes nonintervention strategy
P	Governments that adopt nonintervention strategy will be punished by higher authorities
F	Housing subsidy paid by the society to employees of the unit without participation

**Table 2:** The payoff matrix of the evolutionary game among the government, market, and society.

Strategy combination	(Government, market, society)
(I, P, P)	(*GB*_*M*_+*GB*_*S*_ − *C*_*M*_ − *S*_*M*_ − *C*_*S*_ − *S*_*S*_, *C*_*M*_+*R*_*M*0_+*R*_*M*1_, *C*_*S*_+*R*_*S*0_+*R*_*S*1_)
(I, P, NP)	(*GB*_*M*_ − *C*_*M*_ − *S*_*M*_, *C*_*M*_+*R*_*M*0_+*R*_*M*1_, *R*_*NS*_ − F)
(I, NP, P)	(*GB*_*S*_ − *C*_*S*_ − *S*_*S*_, *R*_*NM*_, *C*_*S*_+*R*_*S*0_+*R*_*S*1_)
(I, NP, NP)	(0, *R*_*NM*_, *R*_*NS*_ − F)
(NI, P, P)	(*GB*_*M*_+*GB*_*S*_+*R*_0_ − P, *R*_*M*0_+*R*_*M*1_, *R*_*S*0_+*R*_*S*1_)
(NI, P, NP)	(*GB*_*M*_+*R*_0_ − P, *R*_*M*0_+*R*_*M*1_, *R*_*NS*_ − F)
(NI, NP, P)	(*GB*_*S*_+*R*_0_ − P, *R*_*NM*_, *R*_*S*0_+*R*_*S*1_)
(NI, NP, NP)	(*R*_0_ − P, *R*_*NM*_, *R*_*NS*_ − F)

**Table 3:** Agent-based model parameters and variable descriptions.

Parameter	Description
*S*_*G*_(t)	The strategic space of government subject
*S*_*M*_(t)	The strategic space of market subject
*S*_*S*_(*t*)	The strategic space of social subject
*N*_*G*_	Number of government agents
*N*_*M*_	Number of market agents
*N*_*S*_	Number of social agents
*N*_*S*_*G*_=1_t	Number of government agents who choose intervention strategy in period *t*
*N*_*S*_*M*_=1_t	Number of market agents who choose participation strategy in period *t*
*N*_*S*_*S*_=1_t	Number of social agents who choose participation strategy in period *t*
*x*(*t*)	Probability of government intervention strategy at period *t*, *x*(*t*) ∈ [0,1]
*y*(*t*)	Probability of market participation strategy at period *t*, *y*(*t*) ∈ [0,1]
*z*(*t*)	Probability of social participation strategy at period *t*, *z*(*t*) ∈ [0,1]
*FR*_*G*(*i*)_(*t*)	The *Agent*_*i*_ in the government subject benefits from the actual combination of strategies in the *t*-period game
*FR*_*M*(*j*)_(*t*)	The *Agent*_*j*_ in the market subject benefits from the actual combination of strategies in the *t*-period game
*FR*_*S*(*k*)_(*t*)	The *Agent*_*k*_ in the social subject benefits from the actual combination of strategies in the t-period game
*IR*_*G*_(*t*)	Expected benefits of government intervention strategy
*NIR*_*G*_(*t*)	Expected benefits of government nonintervention strategy
*PR*_*M*_(*t*)	Expected benefits of market participation strategy
*NPR*_*M*_(*t*)	Expected benefits of market nonparticipation strategy
*PR*_*S*_(*t*)	Expected benefits of social participation strategy
*NPR*_*S*_(*t*)	Expected benefits of social nonparticipation strategy

*Note.* The total number of agents in each agent remains unchanged.

**Table 4:** Eigenvalues of Jacobi matrix corresponding to each equilibrium point.

Equilibrium point	Eigenvalue *λ*_1_	Eigenvalue *λ*_2_	Eigenvalue *λ*_3_
*E*_1_(0, 0, 0)	*P* − *R*_0_	*R*_*M*0_+*R*_*M*1_ − *R*_*NM*_	*R*_*S*0_+*R*_*S*1_ − *R*_*NS*_+*F*
*E*_2_(1, 0, 0)	*R*_0_ − *P*	*C*_*M*_+*R*_*M*0_+*R*_*M*1_ − *R*_*NM*_	*C*_*S*_+*R*_*S*0_+*R*_*S*1_ − *R*_*NS*_+*F*
*E*_3_(0, 1, 0)	*P* − *R*_0_ − *C*_*M*_ − *S*_*M*_	−*R*_*M*0_ − *R*_*M*1_+*R*_*NM*_	*R*_*S*0_+*R*_*S*1_ − *R*_*NS*_+*F*
*E*_4_(0, 0, 1)	*P* − *R*_0_ − *C*_*S*_ − *S*_*S*_	*C*_*M*_+*R*_*M*0_+*R*_*M*1_ − *R*_*NM*_	−(*R*_*S*0_+*R*_*S*1_ − *R*_*NS*_+*F*)
*E*_5_(1, 1, 0)	−(*P* − *R*_0_ − *C*_*M*_ − *S*_*M*_)	−(*C*_*M*_+*R*_*M*0_+*R*_*M*1_ − *R*_*NM*_)	*C*_*S*_+*R*_*S*0_+*R*_*S*1_ − *R*_*NS*_+*F*
*E*_6_(1, 0, 1)	−(*P* − *R*_0_ − *C*_*S*_ − *S*_*S*_)	*C*_*M*_+*R*_*M*0_+*R*_*M*1_ − *R*_*NM*_	−(*C*_*S*_+*R*_*S*0_+*R*_*S*1_ − *R*_*NS*_+*F*)
*E*_7_(0, 1, 1)	*P* − *R*_0_ − *C*_*M*_ − *S*_*M*_ − *C*_*S*_ − *S*_*S*_	−(*R*_*M*0_+*R*_*M*1_ − *R*_*NM*_)	−(*R*_*S*0_+*R*_*S*1_ − *R*_*NS*_+*F*)
*E*_8_(1, 1, 1)	−(*P* − *R*_0_ − *C*_*M*_ − *S*_*M*_ − *C*_*S*_ − *S*_*S*_)	−(*C*_*M*_+*R*_*M*0_+*R*_*M*1_ − *R*_*NM*_)	−(*C*_*S*_+*R*_*S*0_+*R*_*S*1_ − *R*_*NS*_+*F*)

**Table 5:** Parameter values in corresponding scenarios at different evolution stages.

Stage	Scenario		*GB*_*M*_	*GB*_*S*_	*C*_*M*_	*C*_*S*_	*S*_*M*_	*S*_*S*_	*R*_*M*0_	*R*_*M*1_	*R*_*NM*_	*R*_*S*0_	*R*_*S*1_	*R*_*NS*_	*R*_0_	*P*	F
1	1	1-1	5	5	2	1	2	1	3	2	6	2	1	5	5	4	1.5
		1-2	5	5	2	1	2	1	3	2	6	1	1	5	5	4	1
		1–3	5	5	1.5	1	1.5	1	3	1	6	2	1	5	5	4	1.5
		1-4	5	5	1.5	1	1.5	1	3	1	6	1	1	5	5	4	1
2	5	5-1	5	5	1.5	1	1.5	1	3	1	6	1	1	5	2	6	1
		5-2	5	5	1.5	1	1.5	1	3	1	6	1	1	5	1	7	1
3	6	6-1	5	5	1.5	1	1.5	1	3	2	4	1	1	5	2	6	1
		6-2	5	5	1.5	1	1.5	1	3	2	6	1	1	5	2	6	1
		6-3	5	5	1.5	1	1.5	1	3	2	4	1	1	5	1	7	1
		6-4	5	5	1.5	1	1.5	1	3	2	6	1	1	5	1	7	1
	7	7-1	5	5	1.5	1	1.5	1	3	1	6	2	2	5	2	6	2
		7-2	5	5	1.5	1	1.5	1	3	1	6	2	1	5	2	6	1.5
		7-3	5	5	1.5	1	1.5	1	3	1	6	2	2	5	1	7	2
		7-4	5	5	1.5	1	1.5	1	3	1	6	2	1	5	1	7	1.5
4	8	8-1	5	5	1.5	1	1.5	1	3	2	4	2	2	5	1	7	2
		8-2	5	5	1.5	1	1.5	1	3	2	4	2	1	5	1	7	1.5
		8-3	5	5	1.5	1	1.5	1	3	2	6	2	2	5	1	7	2
		8-4	5	5	1.5	1	1.5	1	3	2	6	2	1	5	1	7	1.5

## Data Availability

The data used to support the findings of this study are available from the corresponding author upon request.
